# Diagnostic and prognostic role of circulating neutrophil extracellular trap markers and prekallikrein in patients with high-grade serous ovarian cancer

**DOI:** 10.3389/fonc.2022.992056

**Published:** 2022-12-22

**Authors:** Jisoo G. Kim, Se Ik Kim, Sang Hoon Song, Ja-Yoon Gu, Maria Lee, Hyun Kyung Kim

**Affiliations:** ^1^ Department of Laboratory Medicine, Seoul National University College of Medicine, Seoul, South Korea; ^2^ Department of Obstetrics and Gynecology, Seoul National University College of Medicine, Seoul, South Korea; ^3^ Department of Laboratory Medicine, Seoul National University Hospital, Seoul, South Korea; ^4^ Cancer Research Institute, Seoul National University College of Medicine, Seoul, South Korea; ^5^ Department of Obstetrics and Gynecology, Seoul National University Hospital, Seoul, South Korea

**Keywords:** ovarian cancer, neutrophil extracellular traps, prekallikrein, diagnosis, prognosis

## Abstract

**Objective:**

Tumor-promoting inflammation is among the hallmarks of cancer. Prekallikrein is among the acute-phase reactants in the inflammatory response; moreover, neutrophils release nuclear contents into the extracellular space to create neutrophil extracellular traps (NET). We aimed to investigate the diagnostic and prognostic utilities of circulating plasma NET markers and prekallikrein for high-grade serous ovarian cancer (HGSOC).

**Methods:**

Circulating levels of three NET markers (histone-DNA complex, cell-free DNA, and neutrophil elastase) and prekallikrein were measured in 75 patients with HGSOC and 23 healthy controls. We used an area under the receiver operating characteristic curve (AUC) analysis to investigate their diagnostic and prognostic utilities for HGSOC.

**Results:**

Compared with healthy controls, patients with HGSOC showed significantly higher levels of the three NET markers and prekallikrein. Patients with advanced-stage HGSOC showed significantly higher levels of the cell-free DNA (87.4 vs. 79.5 ng/ml; *P* = 0.013), compared with those with early-stage HGSOC. Further, the levels of histone-DNA complex, neutrophil elastase, and prekallikrein did not significantly differ according to the cancer stage. All markers showed significant diagnostic utility. Notably, a logistic regression-based model that comprised all four markers showed the strongest diagnostic power (AUC, 0.966; 95% confidence interval [CI], 0.933−1.000). Specifically, neutrophil elastase was identified as an independent poor prognostic factor for overall survival (adjusted hazard ratio [aHR], 10.17; 95% CI, 1.09−94.97; *P* = 0.042) and progression-free survival (aHR, 14.47; 95% CI, 1.52−137.35; *P* = 0.020) in patients with HGSOC.

**Conclusions:**

The levels of the three NET markers and prekallikrein might be novel diagnostic and prognostic markers for HGSOC.

## 1 Introduction

Ovarian cancer, one of the most fatal gynecological malignancies, is a global burden. Worldwide, it results in 313,959 new cases and 207,252 deaths annually (1). Given the lack of disease-specific early symptoms and effective screening methods, ovarian cancer tends to be diagnosed at an advanced stage, resulting in relatively high recurrence and mortality rates despite treatment. Early detection and accurate prognostication are essential for improving survival outcomes in patients with ovarian cancer (2). Serum CA-125 levels are used as diagnostic and prognostic biomarkers for ovarian cancer; however, only relying on CA-125 levels is inappropriate for detecting ovarian cancer owing to their low diagnostic performance for early ovarian cancer (3). Therefore, there has been extensive research on novel biomarkers for ovarian cancer.

Tumor-promoting inflammation is among the hallmarks of cancer (4). The inflammatory process is involved in cancer development and progression (5). During inflammation, neutrophils release nuclear contents into the extracellular space, which form structures known as neutrophil extracellular traps (NETs) that can catch microbes (6). NETs are mainly composed of histones and DNA; moreover, neutrophils simultaneously release cytoplasmic enzymes, including neutrophil elastase. NETs interact with cancer cells, which enhances their immune escape and metastatic spread (7). We previously reported that NETs promoted cancer cell migration, invasion, and angiogenesis (8). In literature, circulating NET markers were significantly associated with cancer progression (9). However, few studies have investigated the association of ovarian cancer with NET markers (10–12). Since the tumor microenvironment contains various inflammatory cytokines (13) and the inflammatory process is involved in tumorigenesis and progression of ovarian cancer (14), NET formation could be considerably involved in ovarian cancer.

Inflammation increases the hepatic synthesis of acute-phase reactant proteins, including prekallikrein, which is involved in inflammatory response through the kinin−kallikrein system (15). Patients with cancer have been reported to present with decreased circulating prekallikrein levels due to the high conversion of prekallikrein to kallikrein (16). However, circulating prekallikrein levels may increase due to acute inflammation during ovarian cancer progression.

We aimed to investigate the diagnostic and prognostic utilities of circulating plasma NET markers and prekallikrein for high-grade serous ovarian cancer (HGSOC), which is the most common histologic subtype of ovarian cancer (17).

## 2 Materials and methods

### 2.1 Study population and data collection

We included patients with ovarian cancer according to the following criteria: (1) age ≥18 years ; (2) HGSOC diagnosis between January 2016 and December 2019; (3) having undergone primary debulking surgery (PDS) as well as platinum and taxane-based combination chemotherapy as primary treatment at our institution; (4) having provided informed consent to donate their blood samples for scientific purposes; and (5) having blood samples obtained on the day before surgery and stored at the Seoul National University Hospital (SNUH) Human Biobank. The exclusion criteria included having malignancies other than ovarian cancer; having preoperatively undergone neoadjuvant chemotherapy; loss to follow-up during primary treatment; insufficient data regarding clinicopathological factors; and having severe comorbidities, including uncontrolled diabetes mellitus, end-stage renal disease, or long-term corticosteroid use.

The healthy control group was composed of women who met the following criteria: (1) age ≥ 18 years; (2) no history of the disorder or disease being studied; (3) having provided informed consent for donating blood samples for scientific purposes; and (4) having blood samples obtained at the time of routine health check-up and stored at the SNUH Human Biobank.

In total, 75 patients with HGSOC and 23 healthy women were included in this analysis. We collected baseline characteristics, including age, from the medical records. Additionally, we collected the following clinicopathologic characteristics from patients with HGSOC: International Federation of Gynecology and Obstetrics (FIGO) stage, residual tumor size after PDS, regimens and cycles of postoperative adjuvant chemotherapy, and survival outcomes. Progression-free survival (PFS) was defined as the time interval from the date of initial diagnosis to disease progression according to the Response Evaluation Criteria in Solid Tumors version 1.1 (18). Overall survival (OS) was defined as the time interval from the date of initial diagnosis to cancer-related death or last follow-up.

### 2.2 Measurement of the circulating markers

Peripheral blood samples were collected in sodium citrate tubes (Becton Dickinson, San Jose, CA, USA). After centrifuging the whole blood samples for 15 min at 1550 × g, the plasma was aliquoted and stored at -70°C, with subsequent thawing for further analyses.

The histone*-*DNA complex levels were measured using a cell death detection ELISA kit (Roche Diagnostics, Indiana, USA). Cell-free DNA levels were measured using the Quant-iT PicoGreen dsDNA reagent (Molecular Probes, Eugene, Oregon, USA) and Fluoroskan Ascent microplate fluorometer (Thermo Fisher Scientific Inc., Waltham, Massachusetts, USA). Neutrophil elastase levels were measured using a human neutrophil elastase platinum ELISA kit (eBioscience, Vienna, Austria). Prekallikrein levels were determined using an ELISA kit (Cloud-Clone, Houston, Texas, USA). Initial serum CA-125 levels were evaluated using an immunoradiometric assay kit (Institute of Isotopes, Budapest, Hungary).

### 2.3 Statistical analysis

We performed between-group comparisons for baseline clinicopathologic characteristics and plasma biomarkers. Student’s *t*−test or Mann−Whitney *U* test was used to compare continuous variables, while Pearson’s chi-squared test or Fisher’s exact test was used to compare continuous variables. Among-group comparisons were performed using a one-way analysis of variance, followed by Tukey’s test for pairwise comparisons. Spearman’s rank correlation test was performed to analyze correlations among the levels of individual markers. We conducted receiver operating characteristic (ROC) curve analysis to establish the optimal cutoff values as well as to evaluate the diagnostic utility of each plasma biomarker and their combinations. Kaplan–Meier analysis with log-rank test was used to compare survival outcomes. Regarding multivariate analyses, we constructed Cox proportional hazard regression models to calculate the adjusted hazard ratios (HRs) and 95% confidence intervals (CIs). All statistical analyses were performed using IBM SPSS Statistics version 26.0 (IBM Corp., Armonk, NY, USA), GraphPad Prism version 9.3.0 (GraphPad Software, San Diego, CA, USA), and MedCalc Software version 20.027 (MedCalc Software, Ostend, Belgium). Statistical significance was set at *P* < 0.05.

### 2.4 Ethical statements

This study was approved by the Institutional Review Board of SNUH (No. 2108−119−1245) and was conducted according to the principles of the Declaration of Helsinki and its later amendments.

## 3 Results

### 3.1 Diagnostic values of the circulating markers

**Table 1** summarizes the patient characteristics. Patients with HGSOC were significantly older than healthy controls (mean, 56.1 vs. 38.4 years; *P* < 0.001). Among the 75 patients with HGSOC, 17 (22.7%) and 58 (77.3%) patients had early-stage HGSOC (FIGO stages I–II) and advanced-stage disease (FIGO stages III–IV), respectively. After PDS, 77.3% of the patients with HGSOC achieved complete resection (no gross residual tumor). All patients with HGSOC underwent postoperative adjuvant chemotherapy.

**Table 1:** Characteristics of the study population.

Variables	HGSOC patients (n=75, %)	Healthy controls (n=23, %)	*P* value
Age (years)			
Mean ± SD	56.1 ± 10.7	38.4 ± 5.3	<0.001
Histone−DNA complex (AU)			
Median (IQR)	49.0 (27.0−89.5)	22.0 (19.5−36.0)	0.011
Cell-free DNA (ng/ml)			
Median (IQR)	85.5 (80.3−91.7)	74.6 (69.6−77.7)	<0.001
Neutrophil elastase (ng/ml)			
Median (IQR)	34.1 (29.5−43.5)	18.3 (15.5−39.1)	0.001
Prekallikrein (ng/ml)			
Median (IQR)	8.3 (5.3−12.5)	1.4 (0.0−2.6)	<0.001
CA-125 (U/ml)			
Median (IQR)	849.0 (248.0−2492.0)	13.5 (10.1−15.6)^*^	<0.001
FIGO stage			
I	6 (8.0)		
II	11 (14.7)		
III	45 (60.0)		
IV	13 (17.3)		
Residual tumor after PDS			
No gross residual tumor	58 (77.3)		
<1 cm	8 (10.7)		
≥1 cm	9 (12.0)		
Adjuvant chemotherapy			
Paclitaxel + Carboplatin	66 (88.0)		
Paclitaxel + Carboplatin + Bevacizumab	9 (12.0)		

AU, arbitrary unit; FIGO, International Federation of Gynecology and Obstetrics; HGSOC, high-grade serous ovarian cancer, IQR, interquartile range; PDS, primary debulking surgery; SD, standard deviation.

Missing data: ^*^1.

Compared with healthy controls, patients with HGSOC showed significantly higher levels of histone-DNA complex, cell-free DNA, neutrophil elastase, prekallikrein, and CA-125 levels (**Table 1**). The levels of histone-DNA complexes were significantly correlated with those of cell-free DNA (Spearman’s *rho* = 0.269; *P* = 0.007), neutrophil elastase (*rho* = 0.489; *P* < 0.001), prekallikrein (*rho* = 0.217; *P* = 0.032), and CA-125 (*rho* = 0.232; *P* = 0.022). Additionally, cell-free DNA levels were significantly correlated with neutrophil elastase (*rho* = 0.299; *P* = 0.003) and CA-125 levels (*rho* = 0.457; *P* < 0.001). CA-125 levels were also significantly correlated with neutrophil elastase (*rho =* 0.308; *P* = 0.002) and prekallikrein levels (*rho =* 0.420; *P* = 0.001). No further correlations were observed (**Supplementary Table 1**).

**Figure 1:**
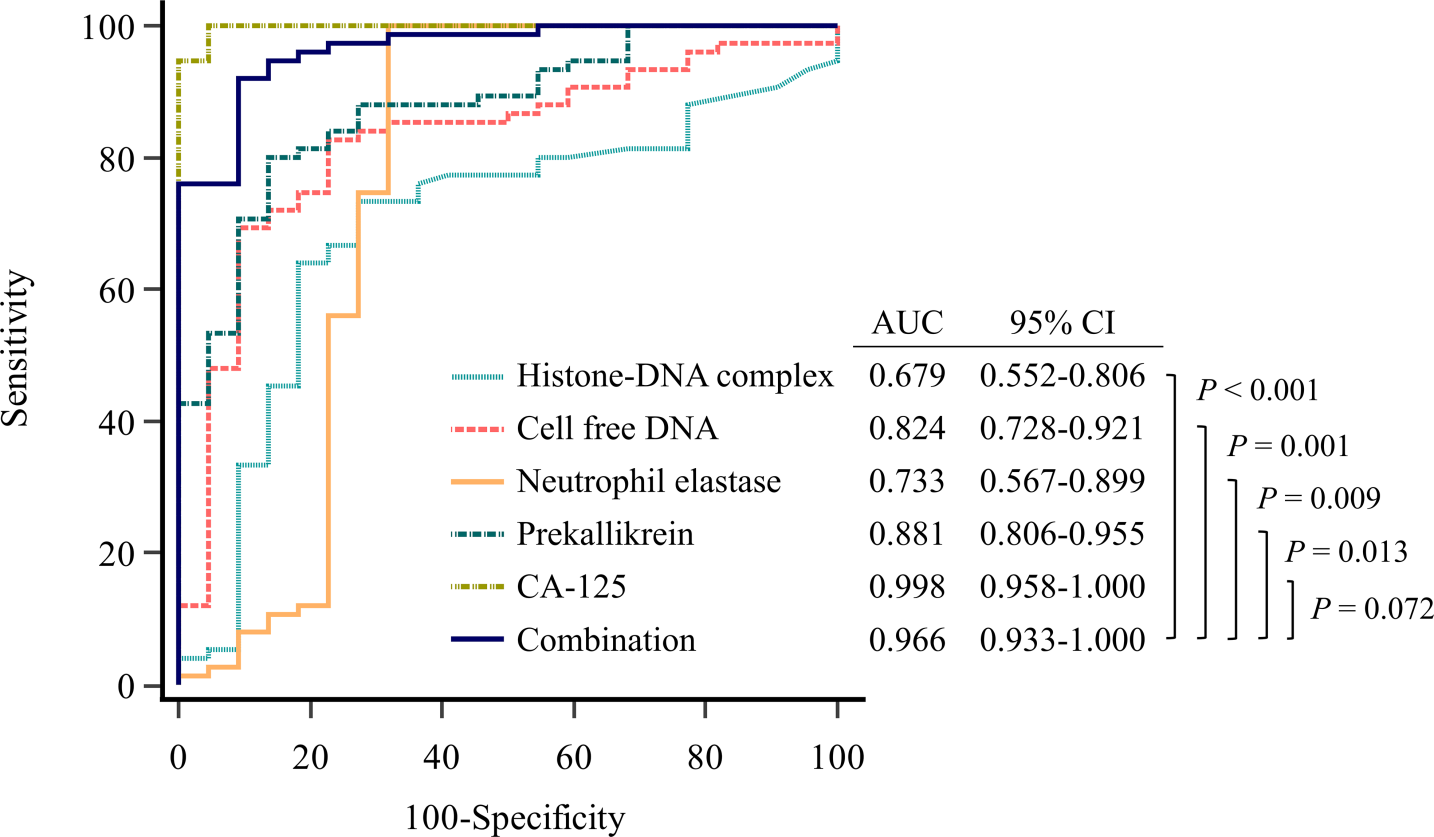
Evaluation of diagnostic performance of the markers for detecting ovarian cancer using receiver operating characteristic curve analysis. Combination marker indicates a logistic regression-based model of histone−DNA complex, cell-free DNA, neutrophil elastase, and prekallikrein.

We performed ROC curve analysis to evaluate the diagnostic utility of the markers for ovarian cancer (**Figure 1**, **Supplementary Table 2**). All four markers showed significant area under the ROC curve (AUC) values in the following order: prekallikrein (0.881; 95% CI, 0.806−0.955), cell-free DNA (0.824; 95% CI, 0.728−0.921), neutrophil elastase (0.733, 95% CI 0.567−0.899), and histone-DNA complex (0.679, 95% CI, 0.552–0.806). In the logistic regression analysis, a combination marker comprising all four markers showed a significantly higher AUC value (0.966; 95% CI, 0.933−1.000) compared with each of the individual markers. The addition of age to this four-marker model further increased the AUC value (0.984; 95% CI, 0.964−1.000). Meanwhile, CA-125 itself had a higher AUC value (0.998; 95% CI, 0.958−1.000) than the four-marker model, but without statistical significance (*P* = 0.072). The sensitivity for ovarian cancer detection was assessed at a fixed specificity of 75%. The four-marker model had the highest sensitivity (97.3%) compared with histone-DNA complex (64.0%), cell-free DNA (82.7%), neutrophil elastase (21.3%), prekallikrein (84.0%), CA-125 (94.7%), and the four-marker plus age model (91.3%).

### 3.2 Prognostic utilities of the circulating markers

**Figure 2:**
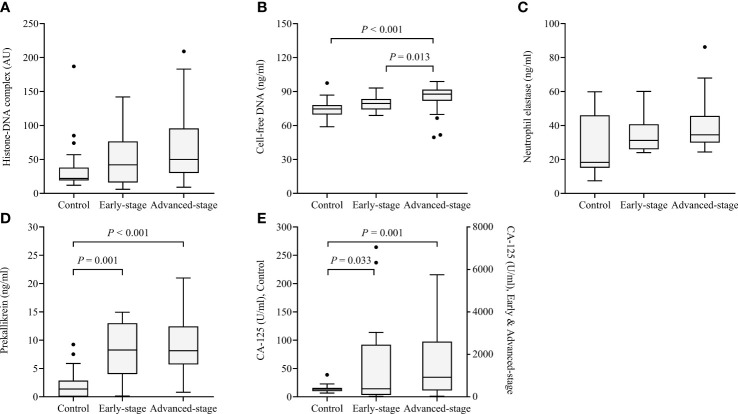
Box and whisker plots of the levels of the four markers in healthy controls (n = 23) as well as patients with early-stage (n = 17) and advanced-stage high-grade serous ovarian cancer (n=58). **(A)** Histone−DNA complex; **(B)** Cell-free DNA; **(C)** Neutrophil elastase; **(D)** Prekallikrein; **(E)** CA-125. The median, range (whiskers), and 25^th^ to 75^th^ percentile (box) are shown.

Histone-DNA complex levels showed a non-significant tendency to increase from the early stage to the advanced stage (**Figure 2**). Cell-free DNA levels were significantly higher in patients with advanced-stage HGSOC than in healthy controls (*P* < 0.001) and patients with early-stage HGSOC (*P* = 0.013). Although neutrophil elastase levels did not differ according to cancer stage, prekallikrein levels significantly increased from healthy controls to patients with early-stage HGSOC (*P* = 0.001) and patients with advanced-stage HGSOC (*P* < 0.001). CA-125 levels were also significantly increased from healthy controls to patients with early-stage HGSOC (*P* = 0.033) and patients with advanced-stage HGSOC (*P* = 0.001).

**Figure 3:**
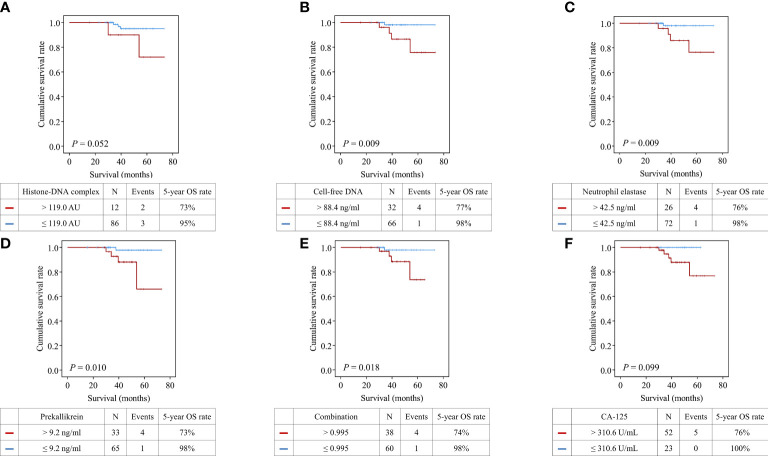
Comparisons of overall survival between patients with high and low levels of the markers. **(A)** Histone−DNA complex; **(B)** Cell-free DNA; **(C)** Neutrophil elastase; **(D)** Prekallikrein; **(E)** Logistic regression-based model of the four markers; **(F)** CA-125.

In survival analysis, patients with high histone-DNA complex levels (> 119.0 AU; n = 12) showed worse OS than those with low histone-DNA complex levels (≤ 119.0 AU; n = 86), with marginal statistical significance (5-year OS rate, 73% vs. 95%; *P* = 0.052) (**Figure 3**). Patients with high cell-free DNA levels (> 88.4 ng/ml; n = 32) had significantly worse OS compared with those with low levels (≤ 88.4 ng/ml; n = 66) (5-year OS rate, 77% vs. 98%; *P* = 0.009). Furthermore, we observed significantly worse OS in patients with high levels of neutrophil elastase (> 42.5 ng/ml; n = 26) (5-year OS rate, 76% vs. 98%; *P* = 0.009), prekallikrein (> 9.2 ng/ml; n = 33) (5-year OS rate, 73% vs. 98%, *P* = 0.010), and combination of the four markers (> 0.995; n = 38) (5-year OS rate, 74% vs. 98%; *P* = 0.018). However, serum CA-125 levels (cutoff value = 310.6 U/mL) did not influence OS (*P* = 0.099).

**Figure 4:**
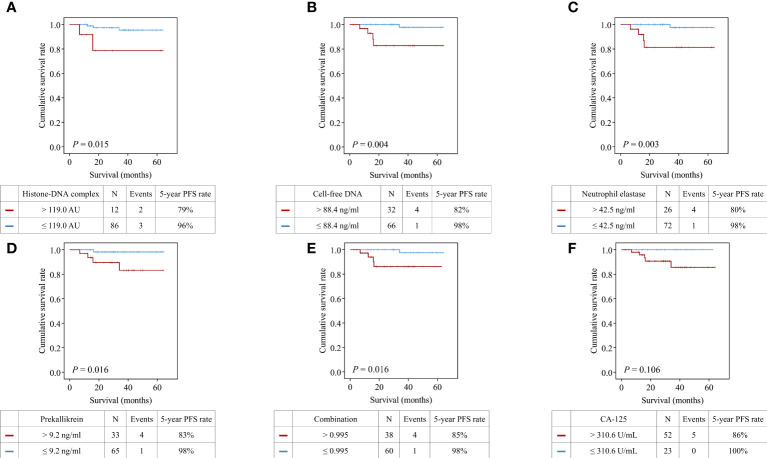
Comparisons of progression-free survival between patients with high and low levels of the markers. **(A)** Histone−DNA complex; **(B)** Cell-free DNA; **(C)** Neutrophil elastase; **(D)** Prekallikrein; **(E)** Logistic regression-based model of the four markers; **(F)** CA-125.

Patients with high histone-DNA complex levels showed significantly worse PFS than those with low levels (5-year PFS rate, 79% vs. 96%; *P* = 0.015) (**Figure 4**). Moreover, we observed significantly worse PFS in patients with high levels of cell-free DNA (5-year PFS rate, 82% vs. 98%; *P* = 0.004), neutrophil elastase (5-year PFS rate, 80% vs. 98%; *P* = 0.003), prekallikrein (5-year PFS rate, 83% vs. 98%; *P* = 0.016), and combination of the four markers (5-year PFS rate, 85% vs. 98%; *P* = 0.016).

Multivariate analyses with adjustment for FIGO stage and postoperative residual tumor revealed that neutrophil elastase was an independent poor prognostic marker for OS (adjusted HR, 10.17; 95% CI, 1.09−94.97; *P* = 0.042) (**Table 2**) and PFS (adjusted HR, 14.47; 95% CI, 1.52−137.35; *P* = 0.020) (**Supplementary Table 3**).

**Table 2:** Factors associated with overall survival in high-grade serous ovarian cancer.

Characteristics	*Univariate analysis*		*Multivariate analysis*											
	HR	*P* (95% CI)	aHR	*P* (95% CI)	aHR	*P* (95% CI)	aHR	*P* (95% CI)	aHR	*P* (95% CI)	aHR	*P* (95% CI)	aHR	*P* (95% CI)
FIGO stage III-IV vs. I-II	27.09	0.518 (0─595838.47)	238456.85	0.982	162636.36	0.979	189380.78	0.979	194097.29	0.979	158847.53	0.979	255052.05	0.979
Residual tumor after PDS ≥1 vs. <1 cm	1.87	0.578 (0.21─16.80)	0.83	0.877 (0.07─9.24)	1.29	0.822 (0.14─12.03)	0.66	0.716 (0.07─6.17)	1.19	0.880 (0.13─10.93)	1.15	0.903 (0.12─10.76)	0.96	0.970 (0.10─8.83)
Histone-DNA complex >119.0 vs. ≤119.0 AU	4.96	0.080 (0.83─29.74)	3.38	0.232 (0.46─24.80)										
Cell-free DNA >88.4 vs. ≤88.4 ng/ml	10.47	0.036 (1.17─94.12)			4.49	0.183 (0.49─40.42)								
Neutrophil elastase >42.5 vs. ≤42.5 ng/ml	10.35	0.037 (1.16─92.68)					10.17	0.042 (1.09─94.97)						
Prekallikrein >9.2 vs. ≤9.2 ng/ml	11.65	0.034 (1.21─112.67)							5.43	0.137 (0.58─50.50)				
Combination^*^ >0.995 vs. ≤0.995	9.38	0.049 (1.01─87.26)									3.07	0.323 (0.33─28.33)		
CA-125 >310.6 vs. ≤310.6 IU/mL	40.55	0.343 (0.02─85562.52)											363682.11	0.971

aHR, adjusted hazard ratio; CI, confidence interval; FIGO, International Federation of Gynecology and Obstetrics; HR, hazard ratio; PDS, primary debulking surgery.

^*^Logistic regression-based model of histone-DNA complex, cell-free DNA, neutrophil elastase, and prekallikrein.

## 4 Discussion

We examined the diagnostic and prognostic utilities of three NET markers (histone-DNA complex, cell-free DNA, and neutrophil elastase) and prekallikrein in patients with HGSOC. All markers showed significant diagnostic power. Furthermore, neutrophil elastase levels were associated with poor survival outcomes in patients with HGSOC.

Malignant tissues are rich in neutrophils, which are susceptible to NET formation (19); moreover, cancer cells can stimulate neutrophils to release NET (7). Accordingly, malignancy is expected to increase circulating levels of NET-formation markers. We previously reported increased circulating levels of NET-formation markers in patients with leukemia or pancreatic cancer (8, 20). However, it remains unclear whether NET markers are increased in patients with ovarian cancer. The present study demonstrated increased levels of three circulating NET-formation markers in patients with ovarian cancer. Neutrophil infiltration is frequently observed in ovarian cancer (11) and NET formation is detected in the omentum of patients with ovarian cancer (21); accordingly, increased levels of NET-formation markers are expected in ovarian cancer.

Moreover, we observed increased circulating levels of prekallikrein in ovarian cancer. Prekallikrein, an acute-phase reactant synthesized in the liver, is a zymogen in the plasma contact system that is involved in coagulation and pro-inflammatory pathways (22). Since inflammatory stimuli induced by continuous ovulation and epithelial cell damage are crucially involved in the initiation and progression of ovarian cancer (13), prekallikrein could be elevated in patients with ovarian cancer. A previous study suggested that prekallikrein levels were decreased in patients with various cancers through its conversion into kallikrein (16). However, we observed increased circulating levels of prekallikrein in patients with ovarian cancer. Ovarian cancer involves an inflammatory microenvironment that can trigger the production of acute-phase reactants (23), which further demonstrates that circulating prekallikrein levels are elevated in ovarian cancer through an acute-phase reaction.

There is increased prekallikrein expression in lung cancer cells (24); additionally, kallikrein-related peptidases are strongly associated with cancer progression (25). Prekallikrein facilitates pathogenic thrombus propagation through the intrinsic coagulation cascade, which may affect ovarian cancer progression (26). In addition, prekallikrein interferes with innate immune signaling and compromises the host defense (27). Therefore, increased prekallikrein expression could be crucially involved in cancer progression.

Patients with ovarian cancer present with increased levels of tumor-associated neutrophils (28). Various proinflammatory cytokines, including cyclooxygenase−2 and interleukin−6, are associated with advanced-stage ovarian cancer (13). Accordingly, the levels of NET markers are expected to increase in patients with advanced-stage ovarian cancer. In our study, cell-free DNA levels were significantly increased in advanced-stage ovarian cancer than in early-stage ovarian cancer. This result is consistent with previous studies which showed a positive correlation between increases in cell-free DNA levels and the stage of ovarian cancer (29). Further, the levels of histone-DNA complex, neutrophil elastase, and prekallikrein did not significantly differ according to the cancer stage; however, their levels showed a tendency of increasing with cancer stage progression. A large-scale study could detect significant differences in the levels of NET markers according to cancer stage.

In our study, histone-DNA complex, cell-free DNA, neutrophil elastase, and prekallikrein showed significant diagnostic power for ovarian cancer, with the combination of these four markers showing the strongest diagnostic value (AUC, 0.966; specificity, 75%; sensitivity, 97.3%). Risk of Ovarian Malignancy Algorithm (ROMA), which combines human epididymis protein 4 (HE4) and CA-125 levels with menopausal status to stratify the risk of ovarian cancer among patients with an ovarian mass, is practically used (30). However, we could not calculate ROMA as we did not investigate study population’s HE4 levels. In order to compare the diagnostic power of our four-marker model and those of ROMA, multi-center cohort studies with a large sample size are warranted.

NET facilitates cancer cell progression and metastasis by promoting cancer cell migration, invasion, and angiogenesis (8). Additionally, NET is involved in immunosuppression and chemoresistance in cancer through its interactions with the tumor microenvironment (31). For instance, the diffusion of doxorubicin, which induces apoptosis through the formation of micro-pores in serous ovarian cancer cells, is reduced by NETs (12).

Accordingly, the elevation of NET markers may lead to a poor prognosis in patients with ovarian cancer. Ascites mitochondrial DNA activates NETs and is associated with a poor prognosis in patients with advanced epithelial ovarian cancer (32), and the lower the nuclear DNA, which can be a surrogate marker for NETs, the better the prognosis in ovarian cancer (33). Also, elevated preoperative plasma total cell-free DNA levels are an independent predictor for poor outcome in patients with epithelial ovarian cancer (34), even who were treated with bevacizumab (35). We examined more NET markers than those previous studies in patients with HGSOC. To our knowledge, this is the first study to show the significant prognostic power of NET markers for ovarian cancer, which could be a useful prognostic indicator for ovarian cancer after validation in a large-scale study.

Notably, our findings indicated that the CA-125 level was not a significant prognostic factor, which is consistent with several reports (36); however, other studies have demonstrated that low CA-125 levels are associated with better OS (37). Moreover, there have been reports that CA-125 is not a useful prognostic factor for low-grade serous (38) or other ovarian cancer (39). Consistent with reports, our findings suggested that the CA−125 level is not a significant prognostic factor for HGSOC.

In addition, we discovered that neutrophil elastase was an independent poor prognostic marker for OS when adjusted for FIGO stage and postoperative residual tumor. The polymorphonuclear neutrophil elastase causes the degradation of E-cadherin, resulting in the loss of polarity and cell contact, leading to a mesenchymal transition and migratory phenotype in ovarian cancer (11).

NETs formed in malignancy carry a negative charge that activates the intrinsic coagulation pathway, which consequently leads to thrombin generation (40). Additionally, histones released from NET induce coagulation activation by increasing coagulation factors in endothelial cells (41). Taken together, NET formation facilitates thrombosis in patients with cancer (42). Therefore, elevated NET formation in patients with ovarian cancer may facilitate thrombosis. Further studies on NET formation as a causative factor of thrombosis in patients with ovarian cancer are warranted.

This study has several limitations. First, this was a single-center retrospective study and our findings should be validated through a large-scale prospective study. Second, the control group only comprised healthy individuals. Future related studies should include both healthy controls and disease controls, including patients with benign ovarian diseases. Third, the patients with HGSOC were older than the healthy controls. Although the relationship between NET formation and aging remains unclear, NET production appears to decrease with age (43). Accordingly, it is unlikely that age differences contributed to increased NET formation in patients with ovarian cancer.

In conclusion, we found that circulating levels of NET markers (histone-DNA complex, cell-free DNA, neutrophil elastase) and prekallikrein were elevated in patients with HGSOC. These markers showed excellent diagnostic performance for HGSOC. Notably, the combination of these markers showed the highest diagnostic power. Moreover, neutrophil elastase was identified as a poor prognostic factor for both OS and PFS. Our findings suggest that NET markers and prekallikrein may be useful diagnostic and prognostic markers for HGSOC. We plan to soon conduct an external validation study.

## Data availability statement

The original contributions presented in the study are included in the article/**Supplementary Material**. Further inquiries can be directed to the corresponding authors.

## Ethics statement

The studies involving human participants were reviewed and approved by Seoul National University Hospital. The patients/participants provided their written informed consent to participate in this study.

## Author contributions

JGK: Collected the related data, contributed to analysis of the data, investigated the study results, and wrote the manuscript. SIK: Collected the related data, investigated the study results, interpreted data, and wrote the manuscript. SHS and J-YG interpreted data and reviewed the manuscript. ML and HKK: Designed the study, investigated and validated the study results, interpreted data, reviewed the manuscript, and approved the final report. All authors contributed to the article and approved the submitted version.
