# Supplementary material for: Diagnostic and prognostic role of circulating neutrophil extracellular trap markers and prekallikrein in patients with high-grade serous ovarian cancer
Source: Front Oncol. 2022 Dec 22;12:992056. doi: 10.3389/fonc.2022.992056 (PMC9813379; doi:10.3389/fonc.2022.992056)
Supplement: Supplementary file 1 [file Table_1.docx]

|  | **Histone-DNA complex** | **Cell-free DNA** | **Neutrophil elastase** | **Prekallikrein** | **CA-125** |
| --- | --- | --- | --- | --- | --- |
| **Histone-DNA complex** | 1 |  |  |  |  |
| **Cell-free DNA** | 0.269^**^ | 1 |  |  |  |
| **Neutrophil elastase** | 0.489^**^ | 0.299^**^ | 1 |  |  |
| **Prekallikrein** | 0.217^*^ | 0.085 | 0.140 | 1 |  |
| **CA-125** | 0.232^*^ | 0.457^**^ | 0.308^**^ | 0.420^**^ | 1 |
| Presented with Spearman's *rho* values. ^*^*P*<0.05; ^**^P<0.01. | | | | | |

**Supplementary Table 1.** Spearman's rank correlation coefficients for the four markers and CA-125
