# Supplementary material for: Diagnostic and prognostic role of circulating neutrophil extracellular trap markers and prekallikrein in patients with high-grade serous ovarian cancer
Source: Front Oncol. 2022 Dec 22;12:992056. doi: 10.3389/fonc.2022.992056 (PMC9813379; doi:10.3389/fonc.2022.992056)
Supplement: Supplementary file 2 [file Table_2.docx]

| **Variables** | **AUC (95% CI)** | ***P* value** | **Cut−off value^*^** | **Sensitivity (95% CI) ^‡^** |
| --- | --- | --- | --- | --- |
| Histone-DNA complex (AU) | 0.679 (0.552−0.806) | 0.006 | >29.0 | 64.0 (52.1−74.8) |
| Cell-free DNA (ng/ml) | 0.824 (0.728−0.921) | <0.001 | >78.0 | 82.7 (72.2−90.4) |
| Neutrophil elastase (ng/ml) | 0.733 (0.567−0.899) | 0.006 | >19.9 | 21.3 (12.7−32.3) |
| Prekallikrein (ng/ml) | 0.881 (0.806−0.955) | <0.001 | >4.4 | 84.0 (73.7−91.4) |
| CA-125 (U/ml) | 0.998 (0.958−1.000) | <0.001 | >22.8 | 94.7 (86.9−98.5) |
| Combination^†^ | 0.966 (0.933−1.000) | <0.001 | >0.7 | 97.3 (90.7−99.7) |
| Combination, Age (years) | 0.984 (0.964-1.000) | <0.001 | >0.8 | 91.3 (72.0-98.9) |
| *The cut−off values were determined as the values which produced the best diagnostic power for ovarian cancer.  †Logistic regression-based model of the four markers: histone-DNA complex, cell-free DNA, neutrophil elastase, and prekallikrein.  ‡Sensitivity was provided at a fixed specificity of 75%.  Abbreviations: AUC, area under the receiver operating characteristic curve; CI, confidence interval. | | | | |

**Supplementary Table 2.** Diagnostic performance of the markers and their combinations for detecting ovarian cancer
