# Supplementary material for: Diagnostic and prognostic role of circulating neutrophil extracellular trap markers and prekallikrein in patients with high-grade serous ovarian cancer
Source: Front Oncol. 2022 Dec 22;12:992056. doi: 10.3389/fonc.2022.992056 (PMC9813379; doi:10.3389/fonc.2022.992056)
Supplement: Supplementary file 3 [file Table_3.docx]

| **Characteristics** | ***Univariate analysis*** | | ***Multivariate analysis*** | | | | | | | | | | | |
| --- | --- | --- | --- | --- | --- | --- | --- | --- | --- | --- | --- | --- | --- | --- |
|  | HR | *P* (95% CI) | aHR | *P* (95% CI) | aHR | *P* (95% CI) | aHR | *P* (95% CI) | aHR | *P* (95% CI) | aHR | *P* (95% CI) | aHR | *P* (95% CI) |
| FIGO stage  III-IV vs. I-II | 33.97 | 0.404  (0.01─132991.41) | 374160.39 | 0.979 | 171676.69 | 0.977 | 255068.18 | 0.973 | 317529.35 | 0.975 | 232067.94 | 0.976 | 203512.66 | 0.975 |
| Residual tumor after PDS  ≥1 vs. <1 cm | 3.38 | 0.287  (0.36─31.65) | 1.53 | 0.727  (0.14─16.39) | 2.80 | 0.384  (0.28─28.32) | 0.76 | 0.813 (0.08─7.39) | 2.97 | 0.364 (0.28─31.0) | 3.36 | 0.312 (0.32─35.12) | 2.56 | 0.430 (0.25─26.28) |
| Histone-DNA complex  >119.0 vs. ≤119.0 AU | 6.96 | 0.035  (1.14─42.41) | 5.78 | 0.069  (0.87─38.44) |  |  |  |  |  |  |  |  |  |  |
| Cell-free DNA  >88.4 vs. ≤88.4 ng/ml | 12.55 | 0.024  (1.39─11.36) |  |  | 6.25 | 0.104  (0.68─57.13) |  |  |  |  |  |  |  |  |
| Neutrophil elastase  >42.5 vs. ≤42.5 ng/ml | 13.14 | 0.021  (1.47─117.87) |  |  |  |  | 14.47 | 0.020  (1.52─137.35) |  |  |  |  |  |  |
| Prekallikrein  >9.2 vs. ≤9.2 ng/ml | 9.25 | 0.047  (1.03─82.98) |  |  |  |  |  |  | 6.48 | 0.097  (0.71─58.86) |  |  |  |  |
| Combination^*^  >0.995 vs. ≤0.995 | 9.30 | 0.047  (1.03─84.13) |  |  |  |  |  |  |  |  | 5.19 | 0.148  (0.56─48.44) |  |  |
| CA-125  >310.6 vs. ≤310.6 IU/mL | 39.68 | 0.351  (0.02─90153.70) |  |  |  |  |  |  |  |  |  |  | 247471.89 | 0.971 |
| Abbreviations: aHR, adjusted hazard ratio; CI, confidence interval; FIGO, International Federation of Gynecology and Obstetrics; HR, hazard ratio; PDS, primary debulking surgery.  ^*^Logistic regression-based model of histone-DNA complex, cell-free DNA, neutrophil elastase, and prekallikrein. | | | | | | | | | | | | | | |

**Supplementary Table 3.** Factors associated with progression-free survival in patients with high-grade serous ovarian cancer
